# Enhancement of antimicrobial properties by metals doping in nano-crystalline hydroxyapatite for efficient biomedical applications

**DOI:** 10.1016/j.heliyon.2023.e23845

**Published:** 2023-12-17

**Authors:** Md. Lawshan Habib, Sanjana Afrin Disha, Md. Sahadat Hossain, Md. Najem Uddin, Samina Ahmed

**Affiliations:** a Department of Applied Chemistry and Chemical Engineering, Faculty of Engineering, Bangabandhu Sheikh Mujibur Rahman Science and Technology University, Gopalganj-8100, Bangladesh; b Institute of Glass and Ceramic Research and Testing, Bangladesh Council of Scientific and Industrial Research (BCSIR), Dhanmondi, Dhaka-1205, Bangladesh; c BCSIR Dhaka Laboratories, Bangladesh Council of Scientific and Industrial Research (BCSIR), Dhaka-1205, Bangladesh

**Keywords:** Hydroxyapatites, Zone of inhibition, Antimicrobial activity, X-ray diffraction, Lattice parameter, Biocompatibility

## Abstract

In this study, we have introduced a method for the synthesis of various metal-doped nano-crystalline hydroxyapatites (HAp) using a standard wet chemical precipitation technique. Both divalent (Ni and Zn) and trivalent (Al and Fe) metals were selected for the doping process. Additional research work was also conducted to assess the antimicrobial efficacy of these doped-HAps against a range of gram-positive and gram-negative microorganisms. All the synthesized metal-doped hydroxyapatite (HAp) exhibited notable antibacterial characteristics against gram-negative bacterial strains, namely *Escherichia coli (E. coli)* and *Salmonella typhi (S. typhi)*, outperforming the pure HAp. The inhibition zone observed for the metal-doped HAp ranged from 14 to 16 mm. The Fe ion displayed a notable inhibitory zone measuring 16 mm, proving to be the most expansive among all tested ions against both *E. coli* and *S. typhi* bacterial strains. The Zn-HAp exhibited a comparable inhibitory zone size of 14 mm against both *S. typhi* and *E. coli*. Additional characterization methods, such as X-ray diffraction (XRD), Fourier transform infrared (FT-IR) spectroscopy, and Scanning electron microscopy (SEM), were used to validate the structural properties of the synthesized metal-doped hydroxyapatite (HAp) samples. The biocompatibility assessment of metal-doped hydroxyapatite (HAp) samples was carried out by haemolysis tests, which revealed that all synthesized hydroxyapatite (HAp) samples have the potential to serve as reliable biomaterials.

## Graphical abstract

**Figure:**
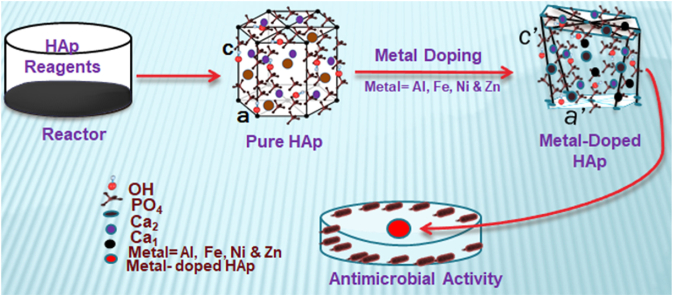

## 1 Introduction

In contemporary times, the eradication of antibiotic-resistant microorganisms has emerged as a formidable challenge for researchers. Henceforth, to tackle antibiotic-resistant microorganisms, innovative methodologies need to be formulated. Researchers are increasingly inclined towards the utilization of metals with antimicrobial properties and metal oxide-doped hydroxyapatite (HAp) as an alternative to mitigate the issues linked to antibiotics and antibiotic resistance. Metal ions engage with microbial membranes to alter their structural properties and permeability, deactivate proteins through binding interactions, and hinder microbial replication by disrupting their nucleic acids [[1], [2], [3], [4], [5]].

Hydroxyapatite, abbreviated as HAp, is a calcium phosphate ceramic represented by the chemical formula Ca_10_(PO_4_)_6_(OH)_2_. It maintains a calcium-to-phosphorus molar ratio of exactly 1.67 and shares both chemical and mechanical similarities with the mineral components present in sturdy anatomical structures such as bone, teeth, enamel, and calcified segments of tendons [6]. HAp displays good biodegradability, bioactivity, biological stability, osteoconductivity, and affinity at biological pH values [7]. It is the most frequently used bioceramic for orthopedic applications due to its similarity to the crystallography and chemical composition of human bone [8,9]. The fundamental advantage of HAp is that it can be manufactured in a variety of forms, including dense ceramic [10], coating material [11], powder [12], and porous material [13,14].

Due to its exceptional versatility, researchers have been drawn to utilizing HAp as a delivery system for proteins, genes, and drugs [[15], [16], [17], [18]]. In addition to serving as a carrier for delivering bioactive components, HAp also holds promise as a ceramic material for applications in bone tissue engineering [19,20], dental applications [21,22], and stem cell differentiation [23,24].

The human bone mineral is a nonstoichiometric nanocrystalline apatite with impurities in the form of co-substituted trace elements of Na, Mg, Fe, Zn, Sr, etc., whereas synthetic HAp typically remains the pure form of HAp with low crystal defects. This is why mechanical strength and bone composition are not perfectly matched by chemically produced HAp. In an effort to replicate the natural bone structure, researchers have conducted experiments involving the incorporation of various elements into the HAp matrix. The effectiveness of an in vivo implant may be influenced by the fact that these metal ions are also essential for protein aggregation [25]. The Ca^2+^ ions in the crystal lattice of HAp can be substituted by different ions such as Al^3+^, Fe^3+^, Zn^2+^, Ni^2+^, Na^+^, etc. to modify various features of synthetic HAp because HAp has a strong ion exchange capacity and a crystal structure composed of Ca^2+^, OH^−^, and (PO_4_)^3-^ [26,27]. HAp enriched with Al decreased crystallinity and resulted in highly anisotropic particles, consequently enhancing the proliferation activity of MG-63 cells [28]. In a separate study, the inclusion of Al in HAp was found to introduce a distorted crystal structure and increased energy states. These characteristics hold significant importance in the field of osteology [29]. Magnetic metal ions such as Fe^3+^ and Co^2+^ were also integrated into HAp nanoparticles and used for MRI, cell separation, targeted medication administration, and hyperthermia [30,31]. Another study verified that the Ni^2+^-doped silicate hydroxyapatite exhibits potent antibacterial properties against *E. coli* and *P. aeruginosa* [32,33]. The zinc ions added to the HAp samples showed an antibacterial impact on *E. coli* and *C. albicans*, which can be used to treat skin infections, microbiologically contaminated water, bone deformities, implant coatings in orthopedic surgery, and for general usage [34].

Our current work is inspired by the fact that it is possible to synthesize monophasic Al, Fe, Ni, and Zn-doped hydroxyapatite nanopowders with high crystallinity. In this paper, we report the effects of various metal-doped HAp composites synthesized by facile chemical synthesis processes, which were examined by several techniques to understand the effect of dopant metals on their structural, morphological, and compositional properties. Furthermore, we assessed the antimicrobial activity of the prepared metal-doped HAp samples against both gram-positive (*Staphylococcus aureus* (ATCC 9144), *Bacillus megaterium* (ATCC 9885)), and gram-negative (*Escherichia coli* (ATCC 11303), *Salmonella Typhi* (ATCC 11311)) bacterial strains and compared their relative antimicrobial activities to develop the most effective composite among the prepared samples.

## 2 Materials and methods

### 2.1 Materials

The experimental reagents employed in this study consisted of ortho phosphoric acid (H_3_PO_4_), calcium hydroxide (Ca(OH)_2_), ammonium hydroxide (NH_4_OH), nitric acid (HNO_3_), ethanol (C_2_H_5_OH), aluminium nitrate nonahydrate Al(NO_3_).9H_2_O, iron (III) nitrate nonahydrate Fe(NO_3_)_3_·9H_2_O, nickel (II) nitrate hexahydrate Ni(NO_3_)_2_·6H_2_O, and zinc nitrate hexahydrate Zn(NO_3_)_2_·6H_2_O. These reagents were procured from Sigma-Aldrich and employed in the experiment without undergoing additional purification. The Institute of Glass and Ceramic Research and Testing Laboratory (BCSIR) provided the deionized (DI) water utilized in all of the experiments.

### 2.2 Synthesis of pure and metal-doped hydroxyapatite (HAp)

The preparation of pure and metal-doped hydroxyapatite (HAp) was conducted using a typical wet chemical precipitation process, as previously documented [35]. In a standard protocol, a solution of 1.67 M Ca(OH)_2_ and a solution of 1.0 M H_3_PO_4_ were prepared in equal volumes for the purpose of synthesizing hydroxyapatite (HAp) while maintaining a Ca/P ratio of 1.67. Deionized water was employed in the process. Phosphoric acid (H_3_PO_4_) was introduced into the calcium hydroxide (Ca(OH)_2_) solution at a rate of 3.0 mL per minute. The reaction was conducted under certain circumstances, including a solution pH range of 10–11, which was achieved by adding ammonium hydroxide (NH_4_OH) solution and at ambient temperature. The reaction was stirred continuously until it reached completion. Following this, a precipitate was formed and separated using filtration, after which it was subjected to drying in an oven at a temperature of 105 °C for 24 h. The entire dry portion was pulverized and then exposed to calcination at a temperature of 900 °C for 30 min. The rate at which the temperature increased throughout the calcination process was 3.5 °C per minute.

The bioceramic samples were manufactured using a similar methodology as that employed for hydroxyapatite (HAp), with the addition of metal dopants. The compounds Al(NO_3_)_3_.9H_2_O, Fe(NO_3_)_3_.9H_2_O, Ni(NO_3_)_2_.6H_2_O, and Zn(NO_3_)_2_.6H_2_O were dissolved in absolute ethanol and subsequently introduced into a solution containing Ca(OH)_2_ and H_3_PO_4_. The molar ratio of (Ca + Al)/P was maintained at a consistent value of 1.67 throughout all samples containing metals. The samples that were produced were labeled as Pure HAp, Al-HAp, Fe-HAp, Ni-HAp, and Zn-HAp, respectively. The metals were doped using wet chemical precipitation method and the percentage of metal was 5.0 % of calcium. Equation (1), (2), (3) outline the chemical processes that took place throughout the synthesis process.

(1) 10Ca(OH)_2_ + 6H_3_PO_4_→ Ca_10_(PO_4_)_6_(OH)_2_

(2) MNO_3_+Solvent→ Metal solution

[M = Al^3+^, Fe^3+^, Ni^2+^, Zn^2+^]

(3) 10Ca(OH)_2_ + 6H_3_PO_4_ + Metal solution→ Metal-doped HAp

### 2.3 Characterization

FT-IR, or Fourier Transform Infrared Spectroscopy, is widely used to detect the existence of different functional groups in organic compounds and sometimes inorganic samples [36]. In this study, we utilized an FT-IR spectrophotometer (IR-Prestige 21, Shimadzu, Japan) equipped with an Attenuated Total Reflectance (ATR) accessory. The instrument had a spectral resolution of 4 cm^−1^ and operated in the wavelength range of 4000-400 cm^−1^. By employing this technique, we were able to clearly identify the distinct characteristic functional groups present in both the pure and metal-doped HAps. The X-ray diffractograms of the prepared samples were obtained utilizing a Rigaku SE XRD apparatus employing a Copper radiation source operating at 40 mA and 50 kV. Within the angular range of 2θ = 5–70°, the X-ray diffraction (XRD) patterns of the powdered samples were obtained using Cu Kα radiation with a wavelength of λ = 1.5406 Å. The data was recorded incrementally with a step size of 0.01. Scanning electron microscopy (SEM) (Model JEOL JSM-7610F) was used to examine the morphologies of the synthetically created pure and metal-doped HAp materials since SEM illustrations allow for the determination of the typical size and shape of the samples that were synthesized. The in vitro antimicrobial activity of the pure and various metal-doped hydroxyapatite (HAp) samples was evaluated against both Gram-positive bacterial strains (*Staphylococcus aureus* (ATCC 9144), *Bacillus megaterium* (ATCC 9885)) and Gram-negative bacterial strains (*Escherichia coli* (ATCC 11303), *Salmonella Typhi* (ATCC 11311)) using the well diffusion method. The inocula of all microorganisms were prepared using freshly cultured broth cultures (Tripton soy broth supplemented with 0.6 % yeast extract – Torlak, Belgrade) that underwent incubation at a temperature of 37 °C.

### 2.4 Infrared spectroscopic analysis (FT-IR)

**Fig. 1:**
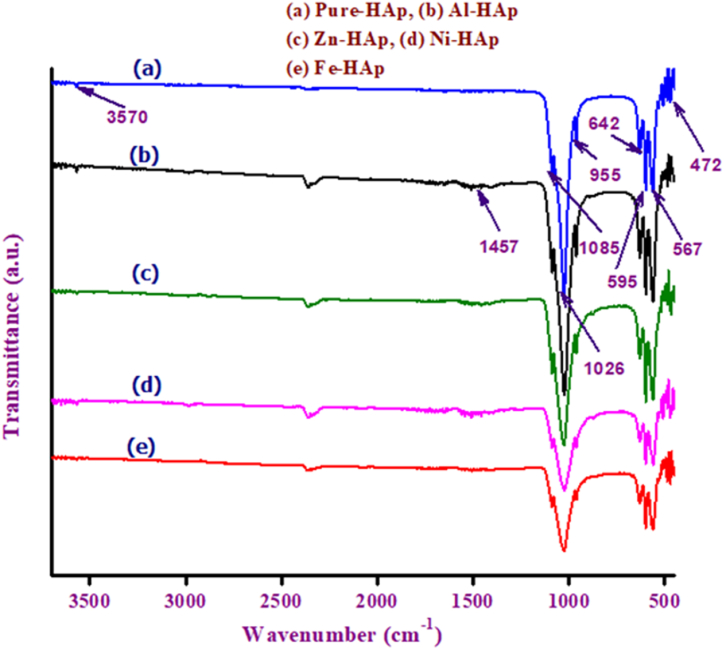
FT-IR spectra of (a) Pure HAp, (b) Al-HAp, (c) Zn-HAp, (d) Ni-HAp, and (e) Fe-HAp.

The Fourier Transform Infrared (FT-IR) spectra for both the pure and metal-doped Hydroxyapatite (HAp) samples are depicted in Fig. 1(a–e).

The hydroxyapatite [Ca_10_(PO_4_)_6_(OH)_2_] molecules consist of phosphate (PO_4_^3−^) and hydroxyl (OH^−^) moieties, which exhibit vibrational activity within the Infrared spectrum. These moieties exhibit distinct peaks at specific wavelengths, irrespective of the nature of the product (amorphous, crystalline, or doped) [36]. As a result of the tetrahedral configuration of the phosphate (PO_4_^3−^) ion, it is anticipated that there will be four distinct vibrational modes, denoted as *ν*_1_, *ν*_2_, *ν*_3_, and *ν*_4_. Notably, in the solid state, two of these vibrations, specifically *ν*_3_ and *ν*_4_, may exhibit splitting due to the influence of the local environment [37]. The discernible vibrational frequencies of the phosphate (PO_4_^3−^) ion are evident across all specimens, exhibiting three stretching modes (1085, 1026, and 955 cm^−1^) and three bending modes (595, 567, and 472) denoted as *ν*_3_, *ν*_4_, *ν*_1_, and *ν*_2_, respectively. The observed peak at 955 cm^−1^ corresponds to the *ν*_1_ symmetric stretching mode, while the peaks at 1085 and 1026 cm^−1^ are indicative of the *ν*_3_ asymmetric stretching of the PO_4_^3−^ moiety. In each and every specimen, the degenerate *ν*_4_ PO_4_^3−^ bending mode manifests itself at the wavenumbers of 595 and 567 cm^−1^, while the *ν*_2_ bending mode is observed at 472 cm^−1^.

The stretching vibration of the hydroxyl group is correlated with the shoulder, approximately at a wavenumber of 3570 cm^−1^. The observed phenomenon could potentially be attributed to the elongation of unbound hydroxyl groups [38], while the lack of wide spectral peaks at 3570 cm^−1^ might be attributed to the annealing process of the samples at a temperature of 600 °C [39]. The vibrational mode associated with the hydroxyl group (OH) can be identified by the absorption band observed at approximately 642 cm^−1^. The residual NO^3−^ moieties originating from the synthesis precursors have been ascribed to the spectral feature observed at 1457 cm^−1^ with analogous peak positions having been documented [38].

### 2.5 X-ray diffraction analysis

**Fig. 2:**
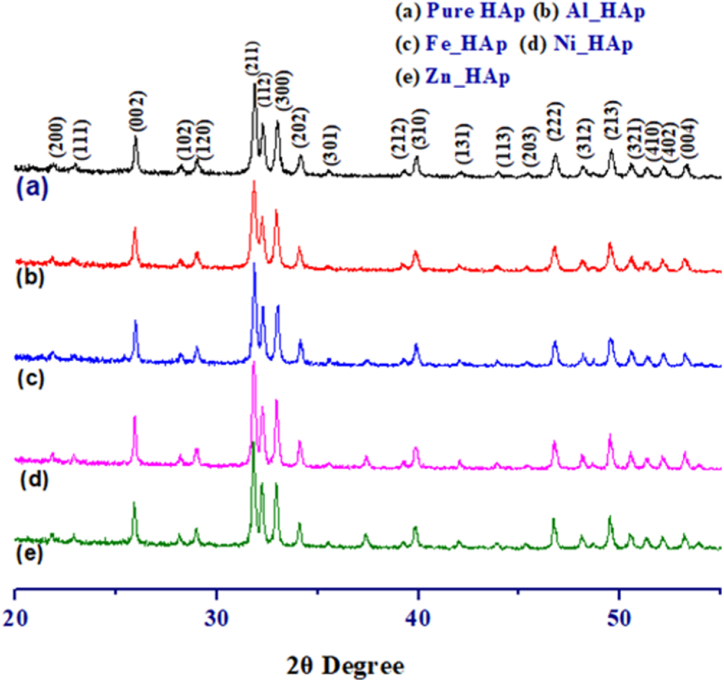
X-ray diffraction patterns of pure and various metals doped HAp. ((a) Zn-HAp (b) Ni-Hap (c) Fe-HAp (d) Al-HAp (e) Pure Hap).

Fig. 2 (a-e) represents the XRD diffractograms of the prepared Pure HAp, Zn-HAp, Ni-HAp, Fe-HAp, and Al-HAp.

Fig. 2 depicts the X-ray diffraction (XRD) diffractograms of the meticulously prepared samples of Pure Hydroxyapatite (HAp), Zinc-doped Hydroxyapatite (Zn-HAp), Nickel-doped Hydroxyapatite (Ni-HAp), Iron-doped Hydroxyapatite (Fe-HAp), and Aluminum-doped Hydroxyapatite (Al-HAp). The 2θ diffraction peaks corresponding to the Hydroxyapatite (HAp) phase, which exhibited excellent agreement with the standard ICDD database (card no. 01-074-0565) for HAp, were observed at angular positions of 25.83779° (002), 31.74018° (211), 32.15124° (112), 32.87631° (300), 34.01908° (202), 39.76592° (130), 46.64704° (222), and 49.42028° (213). These findings provide conclusive evidence for the presence of a hexagonal crystal structure in the sample.

To explore the crystallographic properties of different types of metal-doped hydroxyapatites, various crystallographic parameters were computed, such as the Lattice parameter equation, Degree of Crystallinity, Percentage of HAp, Dislocation density, Crystallite size, Crystallinity index, Microstrain, Percentage of β-TCP, and Volume of the unit cell engaging equations (4)–(12) [40,41]. the value computed for the different types of metal-doped hydroxyapatites, and are registered in Table 1. The lattice parameters and the volume of unit cells were very close to the mentioned standard values of hydroxyapatites. The crystallite sizes computed from the Scherrer equation were within the range of 35–60 nm, and the maximum value was noticed for the Zn-doped hydroxyapatite. The wet chemical synthesis process generates a second phase of β-TCP along with the hydroxyapatites, but in this case no significant peak of β-TCP was noticed, and we computed 0 % of β-TCP using equation (11). The dislocation density and the microstrain were very low for all the synthesized samples, with values lower than 1. The metal doping, dislocation density, and microstrain combined affected the lattice parameters of the synthesized samples.

(4) $$\text{Lattice}\;\text{parameter}\;\text{equation}:{\left(\frac{1}{d_{hkl}}\right)}^{2}=\frac{4}{3}\;\left(\frac{h^{2}+hk+k^{2}}{a^{2}}\right)+\frac{l^{2}}{c^{2}}$$

(5) $$\text{Crystallite}\;\text{size},D_{c}=\frac{K\lambda }{\beta \;\mathrm{Cos}\;\theta }$$

(6) $$\text{Degree}\;\text{of}\;\text{Crystallinity},X_{C}={\left(\frac{K_{a}}{\beta }\right)}^{\;3}={\left(\frac{0.24}{\beta }\right)\;}^{3}$$

(7) $$\text{Percentage}\;\text{of}\;\text{HAp}=\frac{I_{HA\left(121\right)}}{I_{HA\left(211\right)}+I_{\beta -TCP\left(0210\right)}}$$

(8) $$\text{Dislocation}\;\text{density},\delta =\frac{1}{{\left(D_{c\;}\right)}^{2}}$$

(9) $$\text{Crystallinity}\;\text{index},{\text{CI}}_{\text{XRD}=}\sum \frac{H_{\left(202\right)}+H_{\left(300\right)}+H_{\left(112\right)}}{H_{\left(121\right)}}$$

(10) $$\text{Microstrain},\varepsilon =\frac{\beta }{4\;\tan\;\theta }$$

(11) $$\text{Percentage of }\beta ‐\text{TCP}=\frac{I_{\beta -TCP\left(0210\right)}}{I_{HA\left(211\right)}+I_{\beta -TCP\left(0210\right)}}$$

(12) $$\text{Volume}\;\text{of}\;\text{cell},V=a^{2}c\;\sin\;60$$

Where FWHM (in radians) = β = full width at half maxima; Scherrer's constant, K = 0.94; diffraction angle = θ; crystallite size = Dc; lattice parameters are shown as a, b, and c; intensity of the β-TCP at (0210) plane = I_β-TCP(0210)_ and similarly for HAp = I_HAp(0210)_.

**Table 1:** Crystallographic characterization of pure and metals doped hydroxyapatites.

Parameter	HAp	Al-HAp	Zn-HAp	Ni-HAp	Fe-HAp
**Lattice Parameter**, **Å**	a = 9.3998 c = 6.8656	a = 9.4140 c = 6.8798	a = 9.417 c = 6.884	a = 9.414 c = 6.8772	a = 9.4012 c = 6.865
**Crystal Size (nm)**	41.82	35.36	59.69	55.29	48.75
**Degree of Crystallinity**	1.81	1.09	5.62	4.18	2.86
**Crystallinity Index**	1.38	1.44	1.43	1.36	1.41
**Microstrain**	0.17	0.21	0.13	0.13	0.15
**Dislocation density (line/m**^**2**^**)**	0.57	0.79	0.26	0.33	0.42
**Percentage of Hap (%)**	100	100	100	100	100
**Percentage of β-TCP (%)**	0	0	0	0	0
**Volume of Unit Cell (Å**^**3**^**)**	525.35	528.03	528.69	527.44	525.46

Given that cations possessing a diminished ionic radius compared to Ca^2+^ exhibit a predilection for occupying Ca_1_ sites during incorporation, it follows that this phenomenon induces a reduction in dimensions along the a- and c-axes. Conversely, cations endowed with an augmented ionic radius exhibit a preference for substituting Ca_2_ sites [42]. Consequently, the substitution of Ca^2+^ ions with other ions in HAp exhibited a preference for Ca_1_ sites due to the smaller ionic radii of the latter compared to Ca^2+^ [Ca^2+^ = 0.099 nm, Zn^2+^ = 0.074 nm, Ni^2+^ = 0.078 nm, Fe^3+^ = 0.064 nm, Al^3+^ = 0.050 nm]. This was substantiated by the computed lattice parameter values.

### 2.6 Scanning electron microscopy (SEM) studies

**Fig. 3:**
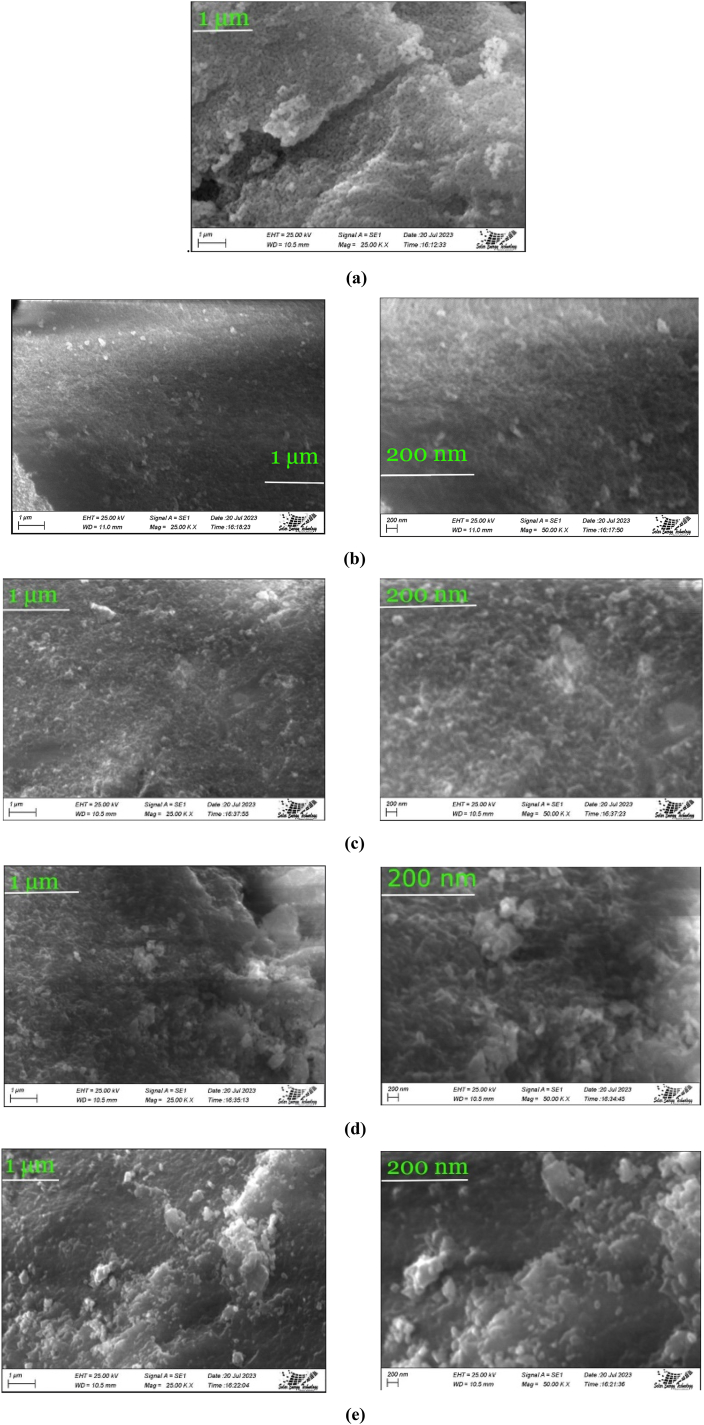
Scanning electron microscopy (SEM) images of pure and metal-doped hydroxyapatite (HAp) samples (a) Pure HAp (b) Al-HAp (c) Fe-HAp (d) Ni-HAp and (e) Zn-HAp.

Fig. 3(a–e) illustrates scanning electron microscope (SEM) micrographs of samples consisting of pure hydroxyapatite (HAp) and hydroxyapatite doped with metal.

Fig. 3 demonstrates micrographs obtained through the utilization of a scanning electron microscope, which effectively captures the visual representation of both pure and metal-doped Hydroxyapatite (HAp) samples. Due to the relatively smaller ionic radii of the calcium ion compared to the cations employed, it is plausible that the doping process of these ions onto the hydroxyapatite (HAp) material has potentially yielded favorable outcomes with respect to the ionic radii of the cation in dispute [43]. The introduction of cations, namely Aluminium, Iron, Nickel, and Zinc, exerted a significant influence on the morphological characteristics of Hydroxyapatite (HAp), as evidenced by the observations depicted in Fig. 3 (b), (c), (d), and (e). It is noteworthy that all the examined samples exhibited agglomerates consisting of finely crystalline structures, exhibiting a remarkable degree of similarity among them [34]. The introduction of Ni and Zn metals into the system led to the formation of HAp flakes, while the incorporation of Fe and Ni revealed the presence of numerous pores on the surfaces of the samples (as depicted in Fig. 3 (c) and (d)). The proposition has been put forth that the nanostructured topography of polymeric scaffolds, previously elucidated, may potentially account for the augmented cellular adhesion and spreading [44]. The potential elucidation of the antibacterial attributes of metal-doped hydroxyapatites (HAps) could plausibly be associated with the diminutive nanoscale dimensions of these HAps. Furthermore, the observed alterations in the material's morphology subsequent to the process of doping were substantiated by the empirical evidence documented in the peer-reviewed scientific literature [38].

### 2.7 Haemolysis test

Three potential classifications of haemocompatibility for a specific sample can be suggested, as outlined in the rules established by the American Society for Testing and Materials (ASTM). The initial criterion pertains to the proportion of observed haemolysis, specifically when it is below 5 %, indicating a high level of haemocompatibility. The second substance exhibits a haemocompatibility level ranging from 5 to 10 %, whereas the third substance demonstrates a haemocompatibility level beyond 20 %, thereby rendering it non-haemocompatible [35].

**Fig. 4:**
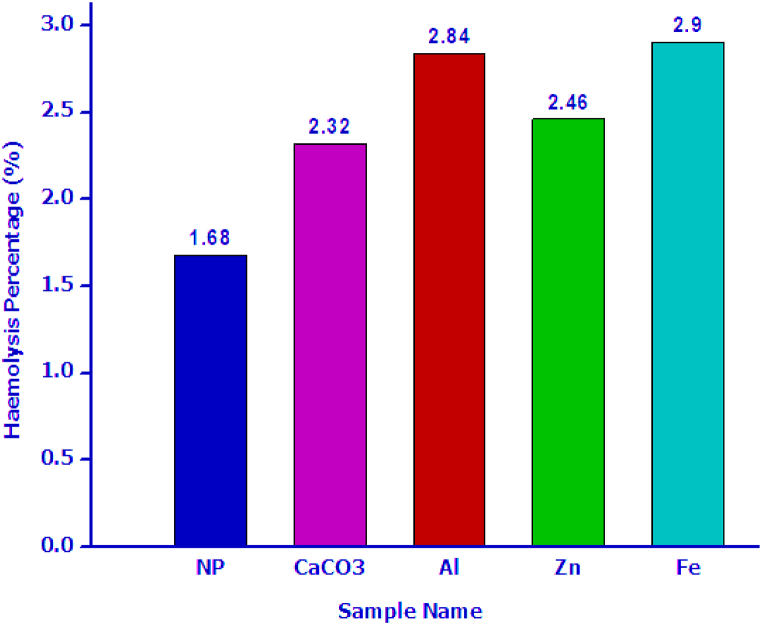
The hemolytic percentage of the pure and metal-doped HAp samples at a dose of 200 μg/mL

The HAp samples, both pure and doped with metals, exhibited exceptional biocompatibility, as evidenced by Fig. 4; specifically, at a concentration of 200 μg/mL, the haemolysis percentage for all samples remained consistently below 3 %. Based on the ASTM standard, it is evident that all synthesized hydroxyapatite (HAP) samples have the potential to serve as reliable biomaterials.

### 2.8 **Antimicrobial activity test (gram-positive:** Staphylococcus *aureus*, *Bacillus megaterium***gram-negative:***Escherichia coli*, *Salmonella Typhi***)**

In order to assess the antibacterial efficacy, a solution of each sample (concentration: 1 mg mL^−1^) was combined with 15 mL of nutrient agar medium. Subsequently, the mixture underwent sterilization at a pressure of 15 lb. per square inch for a predetermined period. The aseptic Petri dish was the designated container for the placement of the sterilized sample. In aseptic conditions, the microorganisms (200 μg/well) derived from the recently cultivated medium of the selected bacteria (diluted 15-fold) were carefully inoculated onto the Petri dish using a glass spreading tool and subsequently incubated overnight. The specimen was subsequently subjected to incubation at ambient temperature and allowed to undergo a 24-h period of maturation. Ciprofloxacin, a fluoroquinolone derivative, was employed as a pharmacological agent with broad-spectrum activity, serving as a positive control antibiotic in the experimental setup. The antimicrobial activity test yielded results through the quantification of the diameter of the zone of inhibition (mm).

**Fig. 5:**
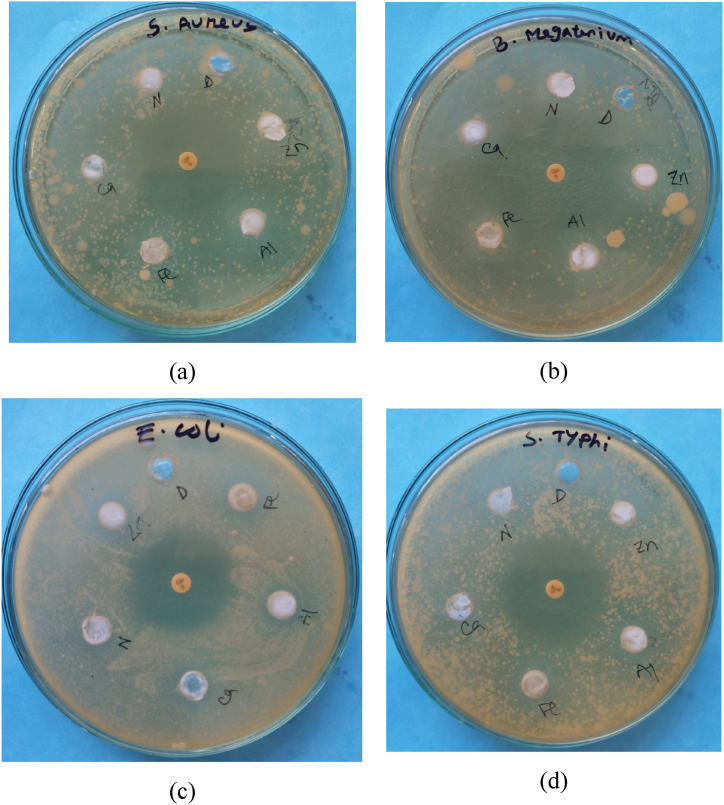
Antimicrobial zones of inhibition of different metal-HAP samples against gram-positive bacteria samples [(a) *S. aureus*, (b) *B. megaterium]* and gram-negative bacteria samples [(c) *E. coli, (d) S. Typhi*)].

**Fig. 6:**
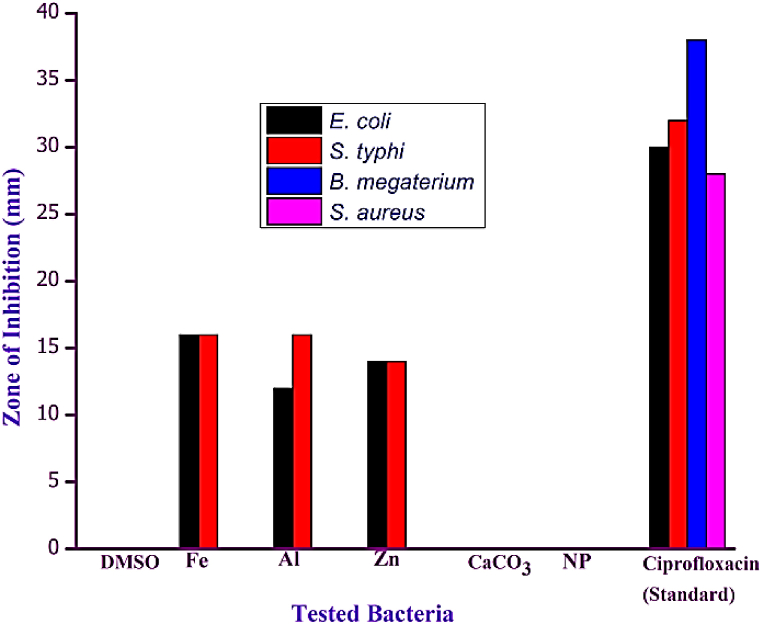
Antimicrobial activity of Fe, Al, and Zn ions substituted with hydroxyapatite and ciprofloxacin as positive controls.

Utilizing the well diffusion technique, a comprehensive investigation was conducted to assess the antimicrobial efficacy of diverse metal-doped hydroxyapatites (HAps) against four distinct bacterial strains (Fig. 5(a–e)). Fig. 6 presents a comparative analysis of the inhibitory zones in Hydroxyapatite (HAp) resulting from the presence of doped ions. The enhanced antibacterial efficacy of pure hydroxyapatite (HAp) nanopowder against *Escherichia coli (E. coli)* and *Salmonella typhi (S. typhi)* was observed upon the incorporation of iron (Fe), aluminium (Al), and zinc (Zn) ions, as depicted in Fig. 5, Fig. 6. The ferrous ion (Fe^2+^) exhibited a substantial inhibition zone measuring 16 mm, surpassing all other tested compounds for both *E. coli* and *S. typhi*, as visually depicted in Fig. 6. The gram-positive bacteria, namely *Bacillus megaterium* (*B. megateriu)* and *Staphylococcus aureus* (*S. aureus)*, did not manifest any discernible inhibitory zones on any of the metal-doped Hydroxyapatite (HAp) substrates, as observed in the experimental results. Al-Hydroxyapatite (Al-HAp) exhibited superior efficacy against the pathogenic bacterium *S. typhi*, as evidenced by the remarkable inhibition zone of 16 mm. In comparison, its effectiveness against *E. coli* was slightly lower, with an inhibition zone measuring 14 mm. The Zn-HAp composite material exhibited a comparable inhibitory radius of 14 mm against both *S. typhi* and *E. coli*. The differential liberation of iron and aluminium from substituted hydroxyapatite (HAp) in comparison to zinc-doped HAp could potentially elucidate the diminished zone of inhibition observed in the latter [45]. In addition, adhesion can promote Fe and Al ion interactions with the cell wall, facilitating their cytotoxic effects.

The findings of this investigation have led to the deduction that none of the hydroxyapatite (HAp) samples doped with metallic elements exhibited susceptibility to gram-positive bacteria, specifically *B. megaterium* and *S. aureus*. One plausible explanation for the observed disparity in sensitivity can be attributed to the inherent dissimilarity in the membranous architectures exhibited by gram-positive and gram-negative bacteria [46]. The samples of Fe-Hydroxyapatite (Fe-HAp) and Al-Hydroxyapatite (Al-HAp) exhibited the most pronounced sensitivity towards *E. coli,* as depicted in Fig. 5. Recent scientific investigations have elucidated the intricate mechanisms governing the antibacterial efficacy of ion-containing compounds. The experimental findings elucidate the intricate interplay between ions and bacterial cell membranes, wherein the ions impede the membranes’ innate functionality, thereby perturbing their structural integrity and culminating in the demise of the cells. Moreover, it is postulated that the intricate arrangement of the extracellular lipid bilayer, pivotal in dictating cellular permeability, subsequent to interaction with the ion-laden antimicrobial substance, unveils a profound disruption in the integrity of the cellular envelope, leading to substantial leakage of intracellular constituents [34]. Through the intricate interplay of electrostatic forces and molecular interactions, the microbial cell adeptly establishes a binding and anchoring mechanism to the cell wall, marking the initial phase of its enthralling interaction with nanoparticles [47]. The specific strain of bacteria and the concentration of ions utilized have been observed to exert discernible influences on the regions of inhibition mediated by Fe-HAp and Al-HAp HAp [44]. Based on the obtained results, it is postulated that the incorporation of ions doped with Hydroxyapatite (HAp) holds promising potential for diverse biomedical implementations. These encompass, but are not limited to, the management of infections in wounds and burns, the utilization of wound dressings, as well as the surface modification of bone prostheses [47,48]. Table 2 represents a comparison between the literatures and present work showing the antimicrobial activity and hemolysis.

**Table 2:** Comparison of antimicrobial activity and hemolysis for hydroxyapatite, and various metal-doped HAp with the previously published works.

Biomaterial Used	Antimicrobial Efficacy				Hemolysis	Refs.
	*S. aureus*	B. megaterium	*E. coli*	S. Typhi		
Hydroxyapatite (Hap)	Insignificant	Insignificant	Insignificant	Insignificant	–	Present Work
Fe-HAp	*-*	*-*	16 mm	16 mm	2.90	
Al-Hap	*-*	*-*	12 mm	16 mm	2.84	
Zn-HAp	*-*	*-*	14 mm	14 mm	2.46	
Hydroxyapatite (Hap)	Insignificant	Insignificant	Insignificant	Insignificant	1.47	[35,40]
Ag-HAP	1 mm	–	4 mm	–	1.4	[49,50]
Cu-HAP	14.5 mm	–	–	–	4.93	[35,51]
Mg-HAP	6.40 mm	–	6.70 mm	–	0.250	[52,53]
Sn-HAp	32 mm	–	–	–	–	[38]
Mo-HAP	22 mm	–	–	–	–	[38]

## 3 Conclusion

The present study introduces a methodological approach for the synthesis of metal-doped hydroxyapatites (HAp) through a conventional wet chemical precipitation process. This method involves the utilization of various antimicrobial metals such as aluminium (Al), iron (Fe), nickel (Ni), and zinc (Zn). The achievement of a homogeneous dispersion of metals within the HAp matrix was successfully accomplished through the implementation of continuous ultrasonication throughout the entire process. This was substantiated by the comprehensive investigations conducted using advanced techniques such as SEM, XRD, and FTIR. The introduction of metallic elements into the HAp framework resulted in a decrease in the level of crystallinity and the formation of metal-doped HAp particles exhibiting a pronounced anisotropic nature. As a result, the antibacterial properties and other desirable characteristics of HAp were found to be significantly improved. The incorporation of metal ions into HAp has been observed to demonstrate noteworthy antibacterial characteristics when tested against gram-negative bacteria, specifically *E. coli* and *S. typhi*. These antibacterial properties have been found to surpass the antimicrobial effectiveness of pure HAp. The observed inhibitory zone for the metal-doped HAp samples exhibited a range of 14–16 mm. The Fe ion demonstrated a notable inhibitory zone measuring 16 mm, surpassing the inhibitory zones observed in the other metal-doped HAp samples. The Zn-HAp material exhibited comparable inhibitory zone diameters of 14 mm when subjected to antimicrobial testing against both *S. typhi* and *E. coli*. The notable antibacterial properties exhibited by metal-doped HAp can be attributed to the formation of a uniform metal (specifically Al, Fe, Ni, and Zn) network within the HAp framework. The aforementioned observation aligns with the outcomes derived from the analyses of metal-doped HAp samples using FTIR, SEM, and XRD techniques. The results obtained from this study suggest that the HAp samples, which were modified with metal dopants, exhibit favorable attributes that may render them a noteworthy material option for diverse biomedical applications. These applications would particularly benefit from materials demonstrating potent antimicrobial activity against gram-negative bacteria. Instances of such applications encompass the utilization of asserted samples as coatings for implants employed in osteogenic surgical procedures, odontological implants, and the management of additional infections instigated by gram-negative bacteria.

## Data availability statement

Data will be made available on request and no data was stored in any publicly available repository.

## CRediT authorship contribution statement

**Md. Lawshan Habib:** Writing – review & editing, Writing – original draft, Supervision, Investigation, Data curation. **Sanjana Afrin Disha:** Methodology, Investigation, Formal analysis, Data curation. **Md. Sahadat Hossain:** Writing – review & editing, Methodology, Formal analysis, Conceptualization. **Md. Najem Uddin:** Formal analysis, Data curation. **Samina Ahmed:** Writing – review & editing, Visualization, Validation, Supervision, Project administration.

## Declaration of competing interest

The authors declare the following financial interests/personal relationships which may be considered as potential competing interests:There is nothing to declare If there are other authors, they declare that they have no known competing financial interests or personal relationships that could have appeared to influence the work reported in this paper.
